# Periodic Fever: A Review on Clinical, Management and Guideline for Iranian Patients - Part II

**Published:** 2014-06

**Authors:** Zahra Ahmadinejad, Sedigeh Mansouri, Vahid Ziaee, Yahya Aghighi, Mohammad-Hassan Moradinejad, Fatemeh Fereshteh-Mehregan

**Affiliations:** 1 Department of Infectious Diseases, Imam Khomeini Hospital; 2 Pediatric Rheumatology Research Group, Rheumatology Research Center; 3 Department of Pediatrics; 4 Children’s Medical Center, Pediatrics Center of Excellence; 5 Growth & Development Research Center, Tehran University of Medical Sciences, Tehran, Iran

**Keywords:** Periodic Fever; Hyper IgD; TRAPS; Autoinflammatory Bone Disorder; Cryopyrin Associated Periodic Syndromes; Sweet’s Syndrome; Blau Syndrome

## Abstract

Periodic fever syndromes are a group of diseases characterized by episodes of fever with healthy intervals between febrile episodes. In the first part of this paper, we presented a guideline for approaching patients with periodic fever and reviewed two common disorders with periodic fever in Iranian patients including familial Mediterranean fever (FMF) and periodic fever syndromes except for periodic fever, aphthous stomatitis, pharyngitis, and cervical adenitis (PFAPA). In this part, we review other autoinflammatory disorders including hyper IgD, tumor necrosis factor receptor–associated periodic syndrome (TRAPS), cryopyrin associated periodic syndromes, autoinflammatory bone disorders and some other rare autoinflammatory disorders such as Sweet’s and Blau syndromes. In cryopyrin associated periodic syndromes group, we discussed chronic infantile neurologic cutaneous and articular (CINCA) syndrome, Muckle-Wells syndrome and familial cold autoinflammatory syndrome. Autoinflammatory bone disorders are categorized to monogenic disorders such as pyogenic arthritis, pyoderma ;gangraenosum and acne (PAPA) syndrome, the deficiency of interleukine-1 receptor antagonist (DIRA) and Majeed syndrome and polygenic background or sporadic group such as chronic recurrent multifocal osteomyelitis (CRMO) or synovitis, acne, pustulosis, hyperostosis and osteitis (SAPHO) syndrome are classified in sporadic group. Other autoinflammatory syndromes are rare causes of periodic fever in Iranian system registry.

## Introduction

In Part I of this paper, we reviewed familial Mediterranean fever (FMF) and periodic fever, aphthous stomatitis, pharyngitis, and cervical adenitis (PFAPA) and repoted on our experience in Iran^[^^1^^]^. In Part II we try to review some other periodic fever syndromes known as autoinflammatory disorders. There are few reports of experience on these disorders in Iran. 

 To establish diagnosis in autoinflammatory disorders is very difficult because there are no definite diagnostic criteria or a gold standard, so it depends on both clinical findings and genetic study to get the diagnosis confirmed (Table 1). All patients usually have a history of repeated hospitalizations and antibiotic therapy. It is due to similar symptoms and signs in autoinflammatory disorders and infectious diseases, immuno-deficiency and/or hematologic disorders. So in each patient, these disorders, especially infectious diseases, should be ruled out^[^^1^^]^. 

***Hyperimmunoglobulin D syndrome***

***(HIDS)***

HIDS or mevalonate kinase deficiency is a hereditary periodic fever syndrome that occurs in unpredictable intervals^[^^2^^-^^4^^]^. Dutch-type periodic fever is another name for this autosomal recessive disorder^[^^5^^]^.

**Epidemiology:** First cases of this syndrome were reported in Dutch population in 1984 but later several patients were reported from other countries and in other races^[^^2^^,^^6^^,^^7^^]^. The syndrome usually starts in childhood and 70% of patients are younger than 1 year old at the beginning of the disease^[^^3^^,^^5^^-^^7^^]^. Sometimes fever attacks are stimu-lated with immunization, trauma, and stress^[^^2^^]^. 

**Pathophysiology:** This syndrome results from a mutation in a gene which is located on chromosome 12q24 and encodes mevalonate kinase (MVK) enzyme^[^^2^^-^^4^^]^. This enzyme contributes to cholesterol synthesis and it has an anti-inflammatory effect^[^^2^^,^^3^^]^. Mutated enzyme has a decreased function with a pro-inflammatory response^[^^2^^,^^8^^]^. 

**Clinical manifestations:** The typical symptoms of the disease are fever, malaise, chills, cervical lymphadenopathy, and diarrhea. Abdominal pain, arthritis or arthralgia, skin rash, and hepatosplenomegaly are other signs^[^^2^^,^^5^^,^^6^^]^. Fever and other symptoms resolve after 3 to 7 days with an afebrile interval lasting 6-8 weeks, but skin rash and arthritis may last longer^[^^5^^,^^8^^,^^9^^]^. Arthritis in HIDS is transient and non-destructive^[^^3^^,^^10^^,^^11^^]^.

**Diagnosis and differential diagnosis:** Diagnosis of HIDS is based on clinical manifestations, elevated serum IgD level (>100), and low activity of MVK enzyme and is confirmed with detection of gene mutation^[^^3^^]^. Immunoglobulin D level should be measured twice with one month interval^[^^7^^]^. Level of IgD might not be very high in children younger than 3 years of age. Immunoglobulin A level in 80% of patients is high^[^^6^^]^.

**Treatment:** There are no absolutely effective drugs for treatment of HIDs, thalidomide, etanercept (anti-tumor necrosis factor), and corticosteroids are used successfully in some patients, but efficacy of these drugs is doubt-ful^[^^12^^-^^14^^]^. Anti interleukine-1 (IL-1) (anakinra or canakinumab) has been effective in severe cases unresponsive to other treatments^[^^10^^]^. 

**Prognosis:** Prognosis of HIDS is excellent even without treatment^[^^6^^]^, and amyloidosis is very rare in these patients^[^^7^^,^^15^^]^. Episodes of fever decreases by aging^[^^16^^]^.

**Hyper IgD in Iran:** In our registry, we reported a HIDS case with concurrent intermittent neutropenia in a 15-month-old boy with good control under intermittent steroid therapy^[^^17^^]^.

***Tumor Necrosis Factor Receptor–Asso-ciated Periodic Syndrome (TRAPS)***

TRAPS or familial Hibernian fever (FHF)^[^^7^^,^^10^^,^^18^^]^, is an autosomal dominant disease that occurs in irregular intervals and is characterized by episodes of fever, pain in muscle groups and painful skin lesions on trunk or extremi-ties^[^^6^^,^^9^^,^^18^^,^^19^^]^.

**Epidemiology: **For the first time, TRAPS was described in a large Irish family, but then it was reported in all countries^[^^7^^,^^18^^]^. The male to female ratio is 3:2^[^^7^^]^ or 1:1^[^^18^^]^ and FHF is more common in Caucasians and Arabs but less common in Indians^[^^20^^]^. This syndrome has been reported in patients 2 weeks to 53 years of age^[^^8^^,^^9^^]^.

**Etiology and Pathophysiology:** The cause of this syndrome is mutation in the gene that encodes the type 1 TNF receptor^[^^6^^,^^8^^,^^18^^,^^19^^]^. The gene is TNFRSF1A and is located on chromosome 12p13.2^[^^3^^,^^18^^,^^19^^,^^21^^]^. TNFR1 is a trans-membrane receptor and the main mediator of signaling by TNF-α^[^^22^^]^. As binding of TNF-α to mutate TNFR1 lessens, as a result, TNF induced apoptosis decreases^[^^3^^]^. Consequently, following increase the activity of TNF, inflammatory response enhances^[^^8^^]^. 

**Clinical manifestations: **Similar to other periodic syndromes, fever is the main symptom in children, in adults attack may be without fever^[^^3^^,^^9^^,^^18^^]^. Fever duration in TRAPS usually is longer than in other periodic fever syndromes such as FMF or in hyper IgD syndrome and it may last up to 3 weeks^[^^2^^,^^7^^,^^18^^,^^21^^,^^23^^,^^24^^]^. 

**Table 1:** Characteristics of classic periodic fever and cryopyrin associated periodic syndromes

**Autoinflmatory disorder**	**Characteristics**
**Hyper IgD ** **syndrome**	Hereditary periodic fever syndromes with good prognosis. Clinical presentation is not specific and diagnosis is based on detection of gene mutation.
**TRAPS**	Recurrent fever with migratory muscle spasms with or without transient blanching erythematous skin lesions. Genetic assessment is necessary for definite diagnosis.
**CINCA or NOMID**	Recurrent fever, relapsing arthritis, urticaria, chronic meningitis and brain atrophy. Anti interlukine-1 drugs control the symptoms effectively
**Muckle-Wells ** **Syndrome**	Fever, limb pain, recurrent urticaria-like lesions, conjunctivitis and deafness. Canakinumab was very effective in patients (complete remission).
**Familial Cold ** **Autoinflammatory ** **Syndrome**	Mildest disease of CAPS, clinical symptoms occur after exposure to cold. Fever, arthralgia, conjunctivitis, non-itching urticarial like rash, and headache. Treatment with anakinra before exposure to cold may be effective in prevention of inflammatory symptoms

IgD: Immunoglobulin D; TRAPS: Tumor Necrosis Factor Receptor–Asso-ciated Periodic Syndrome; CINCA: Chronic Infantile Neurologic Cutaneous and Articular Syndrome; NOMID: Neonatal Onset Multisystem Inflammatory Disease; CAPS: Cryopyrin Associated Periodic Syndromes

Interval between fever episodes has variable frequency but in average it occurs every 5 to 6 weeks^[^^8^^,^^18^^]^. The onset of the disease occurs along with fever and muscular spasm^[^^6^^,^^8^^,^^18^^]^. No known triggers for the onset of the illness are identified^[^^8^^]^. The muscle pain is migratory and affected muscles are tender in palpation and warm^[^^3^^,^^7^^,^^21^^,^^25^^]^. This finding is usually associated with erythematous patches that are warm and tender and are resolved with pressure^[^^2^^,^^3^^,^^9^^,^^18^^,^^25^^]^. Other symptoms of TRAPS are abdominal pain (due to inflammation in peritoneal cavity or muscular cramp in abdominal wall) that is seen in 92% of patients, conjunctivitis or periorbital edema, which is a characteristic for TRAPS and is seen in over 80% of patients, chest pain (in 57% of patients), and arthralgia^[^^3^^,^^6^^-^^8^^,^^18^^,^^19^^,^^24^^]^. Other less common symptoms are arthritis, scrotal pain and lymphadenopathy^[^^3^^,^^7^^]^.

**Laboratory findings:** Increased acute phase reactants such as erythrocyte sedimentation rate (ESR) and C-reactive protein (CRP) and normal muscle enzyme are laboratory findings during episodes but ESR may remain high in intervals^[^^2^^,^^3^^,^^6^^,^^8^^,^^18^^,^^19^^]^. Level of soluble TNFRSF1A may reduce during or between attacks, although normal level does not rule out TRAPS^[^^7^^,^^19^^]^. 

**Diagnosis and differential diagnosis:** Although Alvarez-Lobos et al introduced a diagnostic criteria for TRAPS, it has not been validated in adults or pediatric patients^[^^26^^]^. When the above mentioned symptoms are associated with the family history of similar symptoms and the response to glucocorticoids (but not to colchicine) TRAPS should be considered, but definite diagnosis is made with genetic testing^[^^8^^,^^9^^,^^18^^]^. Nowadays, more than 80 mutations of TNFRSF1A gene have been identiﬁed, but there are several patients with clinical symptoms of TRAPS without specific mutation^[^^8^^,^^9^^,^^18^^]^. FHF should be differentiated from other periodic fevers especially from FMF^[^^3^^,^^9^^,^^18^^]^. Other differential diagnoses are chronic infections and malignancies (lymphoma)^[^^8^^,^^6^^,^^18^^]^.

**Treatment:** Non steroidal anti inflammatory drugs (NSAIDs) are used for treatment of fever but musculoskeletal and abdominal pain do not respond to NSAIDs, so they should be treated with corticosteroids; neither of these drugs can prevent episodes^[^^3^^]^. Etanercept is an anti-TNF agent that decreases severity, duration, and frequency of the attacks^[^^27^^]^. Treatment with anakinra, which is an IL-1 blocker, may be also effective^[^^22^^]^. Treatment with infliximab, an anti-TNF antibody, may lead to paradoxical inflammatory reactions, so it should not be used^[^^27^^,^^22^^]^.

**Prognosis:** The most severe complication of FHF is amyloidosis that occurs in less than 25% of untreated patients and manifests with dysfunction of involved organs^[^^8^^,^^9^^]^. So, prognosis of FHF is related to subsequent amyloidosis^[^^3^^,^^7^^]^. Risk of amyloidosis increases in mutation that affects cysteine amino acids^[^^18^^]^. 

**TRAPS in Iran:** There is no report on TRAPS from Iran in the literature. We have a not reported case under observation in our periodic fever clinic. A 13 year old patient with recurrent fever and abdominal pain and a few patchy erythematous rashes on the trunk and face. Rashes appear with fever and disappear after fever attack. Hyper-IgD and FMF was ruled out with genetic study and trial treatment with colchicine. After starting of low dose oral prednisolone, duration and interval of the febrile attacks decreased significantly. Based on clinical presentation and response to treatment, we suggested TRAPS but genetic study has not been yet performed in this case.

***Cryopyrin Associated Periodic Syndromes (CAPS)***

CAPS refer to a group of diseases which have similar phenotypes all of which result from mutation in the CIAS1 (cold-induced autoinflammatory syndrome) gene, which encodes cryopyrin^[^^28^^]^. This protein regulates generation of pro-inflammatory cytokines^[^^2^^,^^29^^]^. Chronic infantile neurologic cutaneous and articular syndrome (CINCA), Muckle-Wells syndrome (MWS), and familial cold autoinflammatory syndrome (FCAS), which will be explained respectively, belong to CAPS. These syndromes usually have overlapping symptoms the severity of which are affected by other genetic mutations and environmental factors^[28]^. 

 After ruling out infection and malignancies, auto-inflammatory disorders should be considered. However, physician needs to reevaluate each patient with periodic disorder for non-inflammatory syndromes. 

***CINCA Syndrome***

CINCA syndrome is the most severe one among the cryopyrin associated periodic syndromes^[^^30^^]^. These syndromes occur in all ethnic groups without any superiority^[^^15^^]^. CINCA syndrome that is also named neonatal onset multisystem inflammatory disease (NOMID) is a congenital inflammatory disorder that starts in neonatal period and it has various symptoms such as recurrent fever, central nervous system (CNS) involvement, cutaneous manifestations, chronic arthropathy, and morphologic feature^[^^2^^,^^30^^,^^31^^]^. Skin manifestations which are the first presentation at birth in 75% of patients, are similar to urticaria and vary during the day^[^^31^^,^^32^^]^. Urticaria is stimulated with cold. Central nervous system involvement exists in almost all patients and hydrocephalus and ventriculomegaly may be present prenatally^[^^2^^]^. Neurologic manifestations result from chronic meningitis and brain atrophy^[^^30^^,^^32^^,^^33^^]^, and include seizure, sensory organ involvement such as ocular manifestations, progressive sensory neural hearing loss, and headache^[^^3^^,^^31^^,^^32^^,^^33^^]^. Mental retardation exists in some patients^[^^30^^,^^33^^]^ but it is not an initial manifestation^[^^32^^]^. Relapsing joint involvement is also seen in CINCA patients^[^^32^^]^ and severity of arthropathy is related to the age of onset, i.e. patients with a later onset have milder manifestations without destruction^[^^2^^]^. Knees are the most common involved joints and then ankles, feet, and elbows^[^^2^^]^. Patients with CINCA have a typical morphology. They have a short stature, macrocephalus, hoarseness, saddle nose, short extremities, and clubbing^[^^2^^,^^3^^,^^31^^,^^33^^]^. 

 Diagnosis of CINCA is based on the above mentioned specific features^[^^24^^,^^30^^,^^31^^]^. Triad of continuous rash, CNS involvement, and relapsing joint involvement is helpful for diagnosis but this syndrome should be differentiated from other hereditary periodic fever syndromes such as FMF, TRAPS, and HIDS.

 The majority of these syndromes occur sporadically^[^^32^^]^ but there is a mutation in the CIAS1 (now named NLRP3) gene on chromosome 1q44 ^[^^2^^]^ that encodes the cryopyrin protein in 60% of patients^[^^29^^,^^32^^,^^34^^,^^35^^]^. However, this mutation can be found in two other syndromes including MWS and FCAS^[^^3^^,^^7^^,^^21^^,^^30^^]^.

 Treatment with anti IL-1 drugs especially with anakinra is effective in CINCA and results in significant improvement in symptoms of the disease^[^^3^^,^^30^^,^^34^^,^^36^^]^. Nonsteroidal anti-inflammatory drugs and glucocorticosteroids are effective on fever and pain but not on skin lesions ^[^^3^^]^. 

**CINCA in Iran:** Similar to some other rare autoinflammatory disorders, there is no report on CINCA from Iran in the literature. We have 3 suggestive cases to CINCA in our periodic fever clinic. One of them was a 3 month girl with recurrent fever form neonatal period, skin rashes, seizure and hepatosplenomegaly. There was no positive culture for infection. Evaluation for primary immunodeficiency and hemophagocytic lymphohistiocytosis syndrome was negative. After 2 months, she was referred to our clinic. In physical examination, we found a prominent joint. Anakinra was not available in Iranian pharmacopeia. This case was treated with methylprednisolone pulse for 3 days followed by oral prednisolone and ibuprofen. During 1 year follow-up she had 2 fever attacks and she was hospitalized for more evaluation. We did not find any new clinical and paraclinical findings. After 1 year follow-up we tapered prednisolone and now she is on low dose prednisolone (2.5 mg/day) and ibuprofen. Now, she is 18 months old with a small delay in motor development. She had a few episodes of fever and skin rash during last 6 months. 

 Second case was a 6 month old girl referred to our pediatric rheumatology division. She had a history of fever and allergic skin rash from infancy and 2 times admission with osteomyelitis or arthritis suggestion. There was no positive findings for infection but she was treated with antimicrobial agents in the hospital. Urticaria-like skin rashes appeared with fever and disappeared after fever control. We consider autoinflamatory disorders for this patient. Hearing and visual evaluations did not show any significant impairment. She was treated with prednisolone and ibuprofen and she was candidate for treatment with anakinra. After discharge, she had recurrent periodic fever, urticaria-like skin rashes, failure to thrive and motor developmental delay. After 4 months she was treated with anakinra and after 5-10 doses of injection of ankinra, symptoms and signs disappeared. Fever attack was continuing with reduced severity after treatment with ankinra. After 14 months follow-up, she is 20 months old and has attacks of recurrent fever and skin rashes. Acute phase reactants (ESR, CRP) are increased in each attack and reduce after fever control but ESR has not been normal between attacks. Genetic study was performed in our cases, we are waiting for the result.

 Third case was a 3 month old boy with a history of recurrent arthritis and multifocal osteomyelitis. There were no positive serologic findings, positive culture for infection or findings for primary immunodeficiency disorders. Continuous fever, skin rash and arthritis without any response to antibiotic therapy leaded us to autoinflammatory disorders. Based on history and examination and a delay in developmental millstones CINCA was suggested, so he was treated with prednisolone plus ibuprofen and he was candidate for treatment with anakinra. After control of fever, he was discharged. He did not appear for follow-up. Genetic study was not performed in these cases. 

***Muckle-Wells Syndrome (MWS)***

MWS also called urticarial deafness amyloidosis syndrome, is a rare autosomal dominant syndrome^[^^9^^,^^21^^]^. It is of middle severity between CAPS. Episodes of this syndrome are presented with fever, limb pain, recurrent or sub-chronic urticaria-like lesions and conjunctivitis. Episcleritis and optic disc edema are other eye related findings^[^^24^^]^. Fever is usually mild and disappears in 12 to 36 hours^[^^22^^]^. Two-thirds of patients have sensory neural hearing loss that usually starts in the second decade of life^[^^2^^]^. Amyloidosis occurs in one-fourth of patients which can lead to renal failure^[^^36^^]^ and peripheral neuropathy that occurs later than renal amyloidosis^[^^2^^]^. Unlike other CAPS, urticarial rash is not always stimulated with cold^[^^7^^,^^9^^]^. The time of onset is during infancy and sometimes in adolescence. Lifelong arthralgia and non-erosive arthritis may exist^[^^34^^]^. There is a mutation in NLRP3 gene in MWS as in other CAPS, but some mutations are typically found in MWS^[^^7^^,^^35^^,^^37^^]^. Incidence of CAPS syndromes is not subject to sex ^[^^7^^,^^34^^]^. 

 Three anti-IL-1 drugs including anakinra (an IL-1 receptor antagonist), rilonacept (an IL-1 soluble receptor), and canakinumab (a human monoclonal antibody against IL-1β) are effective on CAPS. Canakinumab and rilonacept have FDA approval for treatment of FCAS and MWS^[^^2^^,^^7^^,^^37^^]^. Treatment with canakinumab was very effective and in 96% of patients complete remission was achieved^[^^38^^]^. Studies show that treatment cannot eradicate existing damages but hearing loss may return^[^^2^^]^. 

**Muckle-Wells Syndrome in Iran: **There is no report on Muckle-Wells Syndrome from Iran. 

***Familial Cold Autoinflammatory Syndrome*** ***(FCAS)***

This syndrome, formerly called familial cold urticaria, is the mildest CAPS disease^[^^2^^,^^8^^,^^29^^,^^39^^]^. As other CAPS, FCAS is inherited in an autosomal dominant fashion^[^^9^^]^. FCAS usually begins before 6 months of age^[^^23^^,^^33^^]^ and in 60% of patients symptoms are seen during the first days of life^[^^2^^]^. Clinical symptoms occur within 8 hours^[^^9^^,^^40^^]^ after generalized exposure to cold^[^^33^^]^ and last for 12 to 48 hours^[^^8^^,^^9^^]^. Fever, arthralgia, conjunctivitis, non-itching urticarial like rash, and headache are indicators of FCAS^[^^8^^,^^9^^,^^29^^]^. There are no signs of CNS involvement^[^^28^^]^. Audiology symptoms and deafness is absent^[^^34^^]^. Localized exposure to cold is not a trigger in this disease and it distinguishes FCAS from acquired cold urticaria^[^^33^^]^.

 Treatment with anakinra before exposure to cold may be effective in prevention of inflammatory symptoms^[^^23^^]^. Moving to warm places is recommended to these patients^[^^41^^]^. In all three diseases of CAPS, treatment with anakinra is effective^[^^25^^]^. Rilonacept and canakinumab as long acting IL1 blockers have been approved by FDA for treatment of FCAS and MWS^[^^23^^,^^28^^,^^37^^]^. Long time prognosis of FCAS is good and amyloidosis is uncommon ^[^^9^^]^. 

***Autoinflammatory Bone Disorders***

Autoinflammatory Bone Disorders are a group of autoinflammatory disorders with sterile bone inflammation as a hallmark finding. Some of the disorders of this group are monogenic such as pyogenic arthritis, pyoderma gangraenosum and acne (PAPA) syndrome, the deficiency of IL-1 receptor antagonist (DIRA) and Majeed syndrome (or familial chronic multifocal osteomyelitis). Some other sterile osteitises have polygenic background or are without specific genetic basis, so these disorders are classified as sporadic group,. In which chronic recurrent multifocal osteomyelitis (CRMO) and synovitis, acne, pustulosis, hyperostosis and osteitis (SAPHO) syndrome are also classified^[^^42^^]^. 

***Pyogenic arthritis, Pyoderma gangaenosum and Acne (PAPA) syndrome***

PAPA syndrome is a rare autosomal-dominant disease that characterizes with sterile inflammation in joints and cutaneous manifestation. The cause of the disease is mutation in *PSTPIP1* (proline/serine/threonine phosphatase–interacting protein 1) located on chromosome 15q24-25^[^^34^^]^. This gene produces a protein that mostly exists in hematopoietic cells and regulates T cell activation, cytoskeletal organization and releases of interleukin-1B (IL-1B). Mutation in this gene leads to dysregulation of IL-1B production 77 and macrophages. PAPA syndrome patients have impaired invasive motility 77 and might have dysregulated apoptosis^[^^34^^]^.

 Two major manifestations of PAPA syndrome are erosive arthritis and cutaneous syndromes both of which start in childhood. Joints involvement is recurrent, sterile and destructive and may develop spontaneously or after minor trauma. Skin symptoms of the disease are severe acne, recurrent non healing sterile ulcer called pyoderma gangraenosum and pathergy.

 For treatment, the disease responds well to TNFα blockers such as Infliximab, adalimumab and anakinra. Some new immunosuppressive drugs may also be used to treat PAPA syndrome.

***Majeed Syndrome***

Majeed Syndrome is an autosomal recessive disease caused from a mutation in *LPIN243*. The disease was firstly reported in 1989^[^^9^^]^. Majeed syndrome is a chronic disease which affects the whole life and is characterized by early onset of multifocal osteomyelitis. Other features of Majeed syndrome are congenital anemia and transient inflammatory dermatosis which result from infiltration of neutrophils into dermis and is called Sweet syndrome. Diagnosis is suspected based on clinical manifestations and is confirmed with genetical studies^[^^34^^]^. Treatment of the disease includes non-steroidal anti-inflammatory drugs eventually in combination with corticosteroids or interferon-α.

***Deficiency of the Interlukin-1 Receptor Antagonist (DIRA)***

Deficiency of the IL-1 receptor antagonist is a new TNF-receptor-associated periodic syndrome which was primarily described in 2009^[^^33^^]^. The disease results from mutation in the *IL1RN* (2q14)^[^^27^^]^. It is an autosomal recessive disease. Signs of the disease usually start in prenatal period and include systemic inflammation, multifocal osteolytic lesions, periostitis, and a pustular rash. Other manifestations are heterotopic bone formation around the proximal femur, thrombosis, and rarely vasculitis. Some manifestations which are seen in NOMID but not in DIRA are meningitis, cochlear inflammation, hearing loss, and conjunctivitis or uveitis. DIRA patients have a good response to treatment with the recombinant IL-1 receptor antagonist anakinra^[^^29^^]^. 

**Autoinflammatory Bone Disorders in Iran:** There is no report on autoinflammatory bone disorders with monogenic pattern from Iran. In our experience, a 5 months old boy was referred to our periodic fever clinic. He had a history of soft tissue abscess, arthritis and osteomyelitis after 2 series of vaccination. All evaluation for infection and primary immunodeficiency was negative. Periodic fever, osteomyelitis and prominent articular joints were other symptoms. Visual and hearing evaluation was normal but he had an episode of otitis media with otorrhea. No hepatosplenomegaly or skin rashes were present. The symptoms were compatible with those of auto-inflammatory bone disorders, although we considered CINCA as a diagnosis. Genetic study for DIRA was negative. Further study to rule out/in autoinflammatory disorders is not available now. The symptoms were reduced with prednisolone and Iboprofen, but the disease is not fully controlled. 

***Synovitis, Acne, Pustulosis, Hyperostosis and Osteitis (SAPHO) syndrome or Chronic Recurrent Multifocal Osteomyelitis (CRMO)***

SAPHO and CRMO are chronic nonbacterial or sterile osteomyelitises due to an auto-inflammatory process. Some authorities believe this disorders are different diseases with similar pathway and they have common clinical, radiological and maybe histological findings^[^^43^^,^^44^^]^. Innate immune system is involved and any auto-antibodies or autoreactive T-cells have not been fouind in these disorders. SAPHO is usually described in adult patients with similar symptoms of spondylarthropathies^[^^45^^]^. Vertebral bone is the most common site of involvement and it may lead to vertebral collapse. In SAPHO syndrome, skin involvement such as acne and pustulosis is prominent. CRMO is suggested to be a variant of SAPHO syndrome in children^[^^46^^]^, but in CRMO skin manifestations are rare findings^[^^47^^]^. Exacerbations and remissions of the inflammation is a hallmark for differentiation of these disorders from similar disorders. Increasing number of lesions over the time and self-limiting course without major sequelae are other characteristics. The patients present symptoms of CRMO at school age that include bone lesions. One fourth of the patients had spinal involvement and half of them developed vertebral deformities^[^^29^^]^.

 Treatment is based on NSAIDs and/or corticosteroids. In cases with resistance to these drugs methotrexate, sulfasalazine, and TNF inhibitors are used. CRMO is usually self-limited but a large number of patients experience a prolonged disease^[^^29^^]^. In patients with bone lesions, treatment with bisphosphonates and pamidronate may be helpful and pain resolves 3 months after treatment^[^^48^^]^. MRI is the most sensitive way of diagnosing bone lesions and finding those lesions that are not observed in a simple radiography.

**CRMO in Iranian patients:** There are 2 reports on CRMO from Iran. Both were girls 4.5 and 12 years old. In the latter patient the disease had association with ulcerative colitis^[^^49^^,^^50^^]^. We have a 3.5 years old patient with recurrent osteomyelitis and arthritis of the elbow, wrist and humerus. This case will be published in near future in detail^[^^51^^]^. 

***SWEET’s Syndrome***

In 1964, Sweet described a syndrome with fever and neutrophilic dermatitis^[^^52^^]^. Three types of disease were described by Cohen and Kurzrock^[^^53^^-^^55^^]^. Malignancy-associated form can be associated with hematologic malignancy (such as acute leukemia) and solid tumors. It can be first manifestation of a malignant disorder or first presentation of recurrent malignancy^[109,110]^. Second form is classical form which usually is seen following a respiratory or gastrointestinal infection, pregnancy or rheumatologic disorders (such as irritable bowel disease, systemic lupus erythematosus, Behcet’s disease, FMF and Sarcoidosis)^[^^53^^,^^54^^,^^56^^]^. Third form of Sweet’s syndrome is drug induced following antibiotics (trimethoprim-sulfamethoxazole, nitrofurantoin), NSAIDs (celecoxib and diclofenac), antiepileptics (diazepam and carbamazepine), antihypertensives (hydralazine), oral contraceptives, propylthiouracil and retinoids^[^^53^^,^^56^^]^. 

 Pathogenesis of this syndrome is unknown. Some Cytokines (such as Interleukin 1, TNF-alpha, IL-8, IL-17), human leukocyte antigen serotypes have a possible role in the pathogenesis of this syndrome^[^^57^^-^^59^^]^. The innate immune is involved in this syndrome, so recently it is classified as a autoinflmmatory disorder^[^^57^^]^. 

 Clinical features of this syndrome are a recurrent and acute illness with high fever, peripheral neutrophillia, and skin involvement such as erythematous plaques, erythema nodosum and ulcers. Other manifestations are general malaise, headache, and myalgia, arthralgia, and conjunctivitis^[^^53^^,^^54^^]^. Central nervous system (aseptic meningitis), kidneys, intestines, liver, heart, and lungs may be involved^[^^54^^]^. Diagnostic criteria for classic and drug-induced Sweet syndrome is shown in Table 2. Criteria for malignancy-associated Sweet’s syndrome are similar to classic Sweet’s syndrome.

 In histopathology, diffuse and neutrophil-rich inﬁltrate is seen in the dermis^[^^54^^,^^60^^]^. There is usually no evidence of vasculitis (fibrin deposition or neutrophils) in the vessel wall^[^^61^^]^. 

 There are some reports on successful treatment of Sweet’s syndrome with antibiotics, oral potassium iodide, NSAIDs and colchicines; oral corticosteroids or metylprednolone pulse is very useful in patients with refractory disease^[^^54^^,^^62^^]^. Other treatments are infusion of intravenous immunoglobulin and biologic agents such as anakinra and anti TNF-α agants^[^^62^^]^. 

**Sweet’s syndrome in Iranian patients:** There is a report on 15 adult cases from Iran with classic form of Sweet’s syndrome^[^^63^^]^. All of these patients were female. In our experience, we have a 7 year old boy with recurrent fever, hyper leukocytosis (>40000 in febrile episode) and skin abscess formation or panniculitis. Evaluations for immunodeficiency and leukocyte adhesion deficiency, all were negative. Diagnosis was confirmed by histopathology study. The disease attack was controlled with oral steroid in combination with colchicine. This case is under report^[^^64^^]^. 

**Table 2:** Diagnostic criteria for classic and drug-induced Sweet’s syndrome^[^^65^^]^

**Classical Sweet’s syndrome**
**Major Criteria (both are mandatory)** Abrupt onset of painful erythematous plaques or nodules. Histopathologic evidence of a dense neutrophilic infiltrate without evidence of leukocytoclastic vasculitis.
**Minor Criteria** Pyrexia: T>38°C. Association with an underlying hematologic or visceral malignancy, inflammatory disease, pregnancy, or preceded by an upper respiratory or gastrointestinal infection or vaccination. Excellent response to treatment with systemic corticosteroids or potassium iodide. Abnormal laboratory findings (3 of 4): ESR>20mm/hr / positive CRP / WBC>8000 /Neutrophilia > 70%.
**Drug-induced**
Abrupt onset of painful erythematous plaques or nodules. Histopathologic evidence of a dense neutrophilic infiltrate. Pyrexia: T>38°C. Temporal relationship between drug ingestion and clinical presentation, or temporally related recurrence after oral challenge. Temporally related resolution of lesions after drug withdrawal or treatment with systemic corticosteroids.





Diagnosis of classic Sweet’s syndrome is confirmed by 2 major criteria and two of the four minor criteria but for diagnosis of drug-induced Sweet’s syndrome all five criteria are mandatory.

***Blau Syndrome***

Blau syndrome or “juvenile systemic granulomatosis”^[^^34^^]^ or early onset sarcoidosis^[^^9^^,^^33^^]^ was reported for the first time in 1985^[^^9^^]^. It is a rare disease related to mutation in the *CARD15/NOD2* gene localized on chromosome 16q12^[^^34^^]^. The disease is autosomal dominant and begins in early childhood usually before 4 years of age^[^^9^^,^^35^^]^. 

 The first episode of Blau syndrome is in the first 2 years of age with continuous attack^[^^33^^]^. The most common sites of this syndrome are skin, joints and eyes but not lungs or hilar lymph nodes. Skin lesions which are rarely seen in sarcoidosis in adults include brown colored scaly maculopapules with tapioca-like appearance which are lichenoid-like. Another skin manifestation is erythema nodosum^[^^9^^,^^33^^]^. Destructive arthritis and tendinitis are other manifestations of musculoskeletal involvement^[^^33^^]^. Pneumonitis, eye involvement (uveitis and iritis) and involvement of other organs such as liver and kidney is seen in this syndrome^[^^33^^]^. Characteristic feature of the disease is non-caseating granulomatous lesions involving joints, skin and eyes that lead to symmetric polyarthritis and cataract in 50% of patients. Differentiation of Blau syndrome from sarcoidosis based on histological findings is difficult but clinical symptoms are clearly different^[^^9^^]^. 

 Treatment options for Blau syndrome are corticosteroids, immunosuppressant drugs (such as methotrexate and cyclosporine), infliximab or anakinra^[^^34^^]^. 

**Blau in Iran:** There is no report on Blau syndrome from Iran in the literature and we have no confirmed or suggestive cases with this syndrome in our system registry. 

***What is new in autoinflammatory disorders?***

A new group of autoinflammatory disorders are recently reported with no response to anti IL-1 agents^[^^66^^,^^67^^]^. Three anti IL-1 agents have been approved by FDA for autoinflammatory disorders. Rilonacept and canakinumab are long acting and anakinra is a short acting IL-1 inhibitor^[^^67^^]^. New classification of monogenic autoinflammatory syndromes has been suggested based on completely responsive, variably responsive and unresponsive to anti IL-1 agents (Table 3)^[^^66^^]^. 

 Another classification is based on clinical features of autoinflammatory disorders^[^^67^^]^. In this classification 6 groups of autoinflammatory disorders have been described. The first group is classic periodic fever syndromes that present with recurrent fever, skin rashes and abdominal pain (FMF, TRAPS, HIDS). The second group is cryopyrinopathies that present with neutrophilic urticaria rashes (CINCA, FCAS, Muckle-Wells syndrome). Blau syndrome is authinflammatory syndrome in the third group that presents with granulomatous skin lesions and minimal or low-grade fever^[^^67^^]^. 

 The fourth group is presented with pustular skin rashes and episodic or continuous fever. Autoinflammatory bone disorders (including DIRA, Majeed syndrome), early onset inflammatory bowel disease, deficiency of interleukin 36 receptor antagonist and CARD14-mediated psoriasis are categorized in this group. The fifth group includes poteasome associated autoinflammatory diseases that present with atypical neutrophilic dermatosis and histiocytic-like infiltrate. Autoinflammation disorders with immunodeficiency are the sixth group of autoinflammatory diseases. Autoinflammation and PLCγ 2-associated antibody deficiency (1) and immune dysregulation, PLCγ 2-associated antibody deficiency (2), and immune dysregulation, and HOIL-1 deficiency (3) are 3 known disorders in this group^[^^67^^]^. 

**Table 3:** New classification of autoinflammatory syndromes

**Category **	**Disorder**	**Genetic mutation**
**Responder to IL-1 agents ** **(IL-1 mediated)**	Classic autoinflammatory diseases Familial Mediterranean fever TRAPS HIDS/mevalonate kinase deficiency Monogenic autoinflammatory bone diseases DIRA Majeed syndrome Cryopyrinopathies Familial cold autoinflammatory syndrome Muckle-Wells syndrome NOMID or CINCA	*MEFV *gene *TNFRSF1A* gene *MVK* gene *IL1RN* gene *LPIN2* gene *NLRP3* gene *NLRP3 *gene *NLRP3* gene
**Partially or variable ** **responses to Anti-IL-1 ** **agents**	PAPA Blau syndrome HOIL-1 deficiency APLAID	*PSTPIP1* gene *CARD15/NOD2* gene *HOIL1* *PLCG2*
**Unresponsive to IL-1 (Non-** **IL-1 Mediated)**	Deficiency of IL-36 receptor antagonist PRAAS/CANDLE CARD14-mediated psoriasis Early-Onset IBD	*IL36RN* gene PSMB8 *CARD14* gene *IL10, IL10RA* and/or *IL10RB*

TRAPS: Tumor necrosis factor receptor–associated periodic syndrome; HIDS: Hyperimmunoglobulin D syndrome; DIRA: deficiency of interleukin 1 receptor antagonist; NOMID: Neonatal-onset multisystem inflammatory disease; CINCA: Chronic infantile neurologic cutaneous and articular; PAPA: Pyogenic sterile arthritis, pyoderma gangraenosum, and acne syndrome; APLAID: Autoinflammation and PLCγ 2-associated antibody deficiency and immune dysregulation; PRAAS/CANDLE: Proteasome-associated autoinflammatory syndromes or chronic atypical neutrophilic dermatosis with lipodystrophy and elevated temperature.
